# Intestinal Epithelial Serum Amyloid A Modulates Bacterial Growth In Vitro and Pro-Inflammatory Responses in Mouse Experimental Colitis

**DOI:** 10.1186/1471-230X-10-133

**Published:** 2010-11-10

**Authors:** Erik RM Eckhardt, Jassir Witta, Jian Zhong, Razvan Arsenescu, Violeta Arsenescu, Yu Wang, Sarbani Ghoshal, Marcielle C de Beer, Frederick C de Beer, Willem JS de Villiers

**Affiliations:** 1 Graduate Center for Nutritional Sciences, University of Kentucky, Lexington, USA; 2 Internal Medicine Department, University of Kentucky, Lexington, USA

## Abstract

**Background:**

Serum Amyloid A (SAA) is a major acute phase protein of unknown function. SAA is mostly expressed in the liver, but also in other tissues including the intestinal epithelium. SAA reportedly has anti-bacterial effects, and because inflammatory bowel diseases (IBD) result from a breakdown in homeostatic interactions between intestinal epithelia and bacteria, we hypothesized that SAA is protective during experimental colitis.

**Methods:**

Intestinal SAA expression was measured in mouse and human samples. Dextran sodium sulfate (DSS) colitis was induced in SAA 1/2 double knockout (DKO) mice and in wildtype controls. Anti-bacterial effects of SAA1/2 were tested in intestinal epithelial cell lines transduced with adenoviral vectors encoding the CE/J SAA isoform or control vectors prior to exposure to live *Escherichia coli*.

**Results:**

Significant levels of SAA1/SAA2 RNA and SAA protein were detected by in situ hybridization and immunohistochemistry in mouse colonic epithelium. SAA3 expression was weaker, but similarly distributed. SAA1/2 RNA was present in the ileum and colon of conventional mice and in the colon of germfree mice. Expression of SAA3 was strongly regulated by bacterial lipopolysaccharides in cultured epithelial cell lines, whereas SAA1/2 expression was constitutive and not LPS inducible. Overexpression of SAA1/2 in cultured epithelial cell lines reduced the viability of co-cultured *E. coli*. This might partially explain the observed increase in susceptibility of DKO mice to DSS colitis. SAA1/2 expression was increased in colon samples obtained from Crohn's Disease patients compared to controls.

**Conclusions:**

Intestinal epithelial SAA displays bactericidal properties in vitro and could play a protective role in experimental mouse colitis. Altered expression of SAA in intestinal biopsies from Crohn's Disease patients suggests that SAA is involved in the disease process..

## Background

Serum Amyloid A (SAA) is an acute-phase protein, of which the expression can increase orders of magnitude during infections and stress responses. However, the exact function of SAA is not clear. SAA is evolutionarily strongly conserved, and has been detected in all vertebrates studied to date. Four SAA isoforms have been described in humans and mice [1]. SAA1 and SAA2 represent the main acute-phase isoforms, and are mainly expressed in the liver. SAA3, which is induced during acute and chronic inflammatory responses, is predominantly expressed by macrophages [2]. A fourth isoform, SAA4, is constitutively expressed [3].

SAA structurally resembles an apolipoprotein, and is mainly transported in association with lipoprotein particles, particularly high-density lipoprotein (HDL) [4,5]. During an acute phase response, SAA becomes the main apolipoprotein on HDL, and the displaced Apo-AI [1,5] then becomes available to extract cellular free cholesterol upon interacting with cell-surface ABCA1 [6]. For this reason, and because SAA itself may also extract cholesterol from cells [7,8], it is thought that SAA plays a role in cholesterol metabolism and atherosclerosis [9,10]. Whether SAA is pro- or anti-atherogenic is not yet clear, however, since putative beneficial effects on cholesterol metabolism may be mitigated by effects on inflammation, a know risk factor for atherosclerosis [11].

SAA may affect inflammatory responses by activating its putative receptor on neutrophils (FPRL1), leading to increased production of IL-8 [12]. SAA is also thought to be able to activate TLR2- and TLR4- dependent signaling [13,14]. Recent reports suggest that SAA may also play a role in host defense, notably in the clearance of Gram-negative bacteria. Shah et al. demonstrated that SAA binds to outer membrane protein A of Escherichia coli, which facilitated bacterial clearance by phagocytes [15]. Such a bactericidal effect of SAA is intriguing in light of the reported expression of SAA in intestinal epithelia of rodents [16] and humans [17], since these cells are exposed to many gram-negative bacteria [18]. The intestinal epithelium employs several mechanisms to minimize infiltration and translocation of bacteria, including a mucous layer [19], secretion of immunoglobulin A [20], and the release of an array of anti-microbial proteins and peptides, such as defensins/cryptdins, phospholipases, lysozyme, and Reg III-gamma [19,21-24]. Failure to properly induce and maintain these defense mechanisms, e.g. through defects in sensing mechanisms for bacterial products, may increase the risk for inflammatory bowel diseases (IBD) [25,26]. It is therefore important to characterize the entire spectrum of anti-microbial mechanisms deployed by intestinal epithelial cells.

We hypothesized that SAA would be protective in experimental colitis, by aiding in the killing of Gram- negative bacteria. To test this hypothesis, we generated double knockout (DKO) mice in which the genes encoding the two major acute phase isoforms, SAA1 and SAA2, were inactivated. These mice were challenged with DSS in their drinking water to induce acute colitis. We also tested whether over-expression of SAA in cultured enterocytes would reduce the viability of co-cultured E. coli. In this report, we confirm epithelial expression of SAA in wildtype mice and present evidence that DKO mice are more susceptible to DSS-colitis. Intestinal epithelial SAA strongly reduced the viability of co-cultured *E. coli in vitro*. Acute phase SAA expression was increased in intestinal biopsies of Crohn's Disease patients. Thus, SAA may represent a novel factor in intestinal-epithelial immune homeostasis.

## Methods

### Animals

C57BL/6J mice were purchased from The Jackson Laboratory (Bar Harbor, Maine, USA) for histological examination of SAA expression. The generation of SAA1/SAA2 double knockout mice is described elsewhere in the Methods section. All animals were maintained on a 12-hour light/12-hour dark cycle under specific pathogen-free conditions. The mice had free access to standard rodent diet and water. Frozen intestinal tissue samples from germfree mice (Swiss Webster) were ordered from Taconic. All animals were handled in strict accordance with good animal practice as defined by the relevant national and local animal welfare bodies, and all animal work was approved by the Institutional Animal Care and Use Committee of the University of Kentucky.

### In situ hybridization

C57Bl/6 mice were euthanatized by CO_2 _asphyxiation. Colons and small intestines were frozen in "Optimum Cutting Temperature" (OCT) mounting medium on dry ice, sectioned (16 μm thickness) with a cryostat at -16°C, mounted on Superfrost/Plus slides (Fisher Scientific, Vernon Hills, IL), dried at 37°C, and processed for in situ hybridization. Briefly, sections were washed with phosphate-buffered saline, quickly dehydrated in a series of solutions with increasing ethanol content, and de-lipidated in chloroform. Sections were hybridized overnight in a humid chamber at 55°C using radiolabeled [^35^S] probes (10^6 ^dpm/60 μl) in hybridization buffer (20 mMTris-HCl, pH 7.4, 1 mM EDTA, 300 mM NaCl, 50% formamide, 10% dextran sulfate, 1 × Denhardt's solution). The slides were washed four times for 5 min each in 4× standard saline citrate (SSC) containing 1 mM DTT. To remove single-stranded RNA, sections were washed for 30 min at 37°C in a solution containing RNase A (20 μg/ml). Slides were then washed for 5 min each at room temperature in solutions containing decreasing amounts of SSC (2×SSC, 1×SSC and 0.5×SSC; all with 1 mM DTT), and then washed two times for 30 min at 65°C in 0.5×SSC/1 mM DTT. Sections were cooled to room temperature and dehydrated in ethanol. Slides were then dipped in undiluted Kodak NTB2 emulsion, exposed for five weeks, developed, and then counterstained with Giemsa.

### Probe preparation

Riboprobes were prepared using [^35^S]-UTP and the Maxiscript kit (Ambion, Austin, TX) according to the manufacturer's directions. The following templates were used: Mouse SAA1 and SAA2, a fragment of cDNA (GenBank Accession No. M11130) corresponding to nucleotides 388-606 of the coding sequence. Mouse SAA3, a fragment of cDNA (GenBank Accession No. BC055885) corresponding to nucleotides 376-529 of the coding sequence. [^35^S]-UTP labeled antisense and sense probes were generated by T7 and T3 RNA polymerase using linearized templates, respectively.

### Immunostaining and immunoblotting

Colon sections were fixed in 4% paraformaldehyde in phosphate-buffered saline (PBS), washed in PBS, and incubated in 1% bovine serum containing 0.6% Triton X-100. Slides were incubated overnight with rabbit polyclonal antiserum against purified mouse SAA (generated by Lablogix corporation), washed, and incubated with fluorescein-isothiocyanate (FITC)-conjugated secondary antibody (Jackson ImmunoResearch Laboratories, West Grove, PA). Slides were washed with PBS, mounted in anti-fade mounting medium (Vectashield, Vector Laboratories, Burlingame, CA), and viewed with an epifluorescence microscope (model BX50; Olympus Optical, Melville, NY) equipped with a cooled charge-coupled device camera. Images were digitally acquired using MagnaFire 2.1A software, and recompiled in Adobe Photoshop, version 5.0. Sections stained with secondary antibody alone did not show reactivity (data not shown). Extracts of feces isolated from the ascending colon were prepared by adding radio-immunoprecipitation assay buffer (RIPA) to freeze-dried pellets. Boiled and reduced fecal extracts and samples from conditioned medium of adenovirus-treated epithelial cells were subjected to SDS-polyacrylamide gel electrophoresis, transferred to PVDF membranes, incubated with rabbit anti-SAA, and developed after incubation with horseradish peroxidase conjugated goat-anti rabbit IgG.

### Generation of SAA1 and SAA2 double knockout (DKO) mice

Targeted deletion of both mouse acute phase SAA genes *Saa1 *and *Saa2 *was performed by InGenious Targeting Laboratory Inc. (Stony Brook, NY) using embryonic stem cells derived from C57BL/6 × 129 SVEV mice. The targeting vector contained a neo cassette that replaced ~ 10.1 kb of the *Saa1 *and *Saa2 *genes, which included exon 2 of both oppositely orientated genes. The knockout mice are currently being backcrossed into C57Bl/6 mice, and the same is done with a parallel cohort of C57BL/6 × 129 SVEV mice. Mice from similar generations of both groups of mice, i.e. DKO mice and C57Bl/6 × 129 SVEV mice as control, were used for DSS colitis experiments.

### Induction of colitis

Colitis was induced in 10 weeks old male DKO mice or controls, by adding 3% dextran-sodium sulfate (DSS; molecular weight 40,000 Da; ICN Biomedicals, Aurora, Ohio, USA) to the drinking water, for 7 days. Controls received untreated water. The mice were examined on day 9 for weight loss, colon length, and blood hematocrit (HCT). Paraffin sections of the colons were microscopically analyzed and scored with a histological disease index, as described previously [27]. All procedures using animals had been reviewed and approved by the Institutional Animal Care and Use Committee of the University of Kentucky, and were performed according to the criteria outlined by the NIH.

### Real-time PCR of mouse colon samples

RNA from colon samples from mice treated with DSS were reverse transcribed into cDNA, which was then analyzed for the expression of TNFalpha and osteopontin (a pro-inflammatory cytokine which is associated with DSS colitis [28]) relative to GAPDH by real-time PCR using the following primer pairs: GGC-AGG-TCT-ACT-TTG-GAG-TCA-TTG/GTT-AGA-ACA-CAG-ACT-GG (TNFalpha), AGC-AAG-AAA-CTC-TTC-CAA-GCA-A/GTG-AGA-TTC-GTC-AGA-TTC-ATC-CG (osteopontin), CCA-GGT-TGT-CTC-CTG-CGA-CTT/CCT-GTT-GCT-GTA-GCC-GTA-TTC-A (GAPDH).

### Cell culture studies

CMT93 (a murine rectal epithelial cell line) and HT29 (a human colonic adeno-carcinoma line) intestinal epithelial cells were purchased from American Type Culture Collection (ATCC; Manassas, VA) and were cultured in 4-(2-hydroxyethyl)-1-piperazineethanesulfonic acid (HEPES-) and bicarbonate-buffered Dulbecco's Modified Eagle Medium (DMEM), supplemented with 10% heat-inactivated fetal calf serum and with antibiotics (penicillin, streptomycin and amphotericin), in a 37°C incubator with 5% CO_2 _and 100% humidity. When ~80% of the tissue culture plates were covered with cells, the cells were detached with 0.25% Trypsin/EDTA for sub-culturing or seeding in 12 well tissue culture plates at ~200,000 cells/cm^2^. Confluent monolayers were incubated in absence of antibiotics with adenoviral vectors encoding SAA (AdSAA;) or a control vector (Adnull), both at a multiplicity of infection of ~20. The origin and creation of the adenoviral vectors, with AdSAA encoding the *Saa1/2 *fusion gene of the CE/J mouse strain (producing SAA2.2 protein), has been described elsewhere [29]. The cells were incubated for 48 h to allow for SAA expression. To study regulation of SAA1/2 and SAA3 expression in CMT93 cells, confluent monolayers were incubated with various amounts of E. coli LPS (0111:B4; obtained from Sigma Aldrich) for 16 h. RNA was isolated and transcribed into cDNA prior to real-time PCR analysis with the following primer pairs (5' - 3'): CTG CCT GCC AAA TAC TGA GAG TC/C CAC TTC CAA GTT CCT GTT TAT TAC (SAA1/2), GCT GGC CTG CCT AAA AGA TAC TG/G CAT TTC ACA AGT ATT TAT TCA GC (SAA3), CCA GGT TGT CTC CTG CGA CTT/CCT GTT GCT GTA GCC GTA TTC A (GAPDH). Melting curves were obtained after PCR to assess amplicon quality.

### Bacterial viability assay

A growth curve of *E. coli *DH5α in Luria Broth (LB) was generated by correlating light absorption at 600 nm with the number of colony forming units observed after plating bacterial dilutions on LB agar. On the morning of the experiment, a culture was started and bacteria were allowed to grow until mid-log phase. A total number of bacteria corresponding to a 100× excess compared to intestinal epithelial cells was pelleted, washed three times in PBS, and added as 10× concentrated suspension to the epithelial cells in their original, adenovirus-containing antibiotics-free medium. The epithelial cells were incubated with bacteria for 2 h, at 37°C. Bacteria were also added to a set of wells lacking epithelial cells to determine 100% bacterial viability. After the incubation, serial dilutions of supernatant were spread on LB-agar, and colonies were enumerated after overnight incubation at 37°C.

### RT-PCR of human colon samples

RNA from biopsies from inflamed and non-inflamed ("uninvolved") areas of the terminal ileum and colon of Crohn's Disease patients (n = 10) and of controls with no Crohn's Disease (n = 5) were reverse transcribed into cDNA, which was then analyzed for the expression of SAA1/2 RNA relative to GAPDH RNA by real-time PCR using primer pairs GTA-GGC-TCT-CCA-CAT-GTC-CC/TGG-TTT-TCT-GCT-CCT-TGG-TC and GAA-GGT-GAA-GGT-CGG-AGT-CAA-C/CAG-AGT-TAA-AAG-CAG-CCC-TGG-T, respectively. The samples were obtained with permission of the Institutional Review Board of the University of Kentucky.

## Results

### Expression of acute-phase SAA in mouse intestinal epithelium

**Figure 1:**
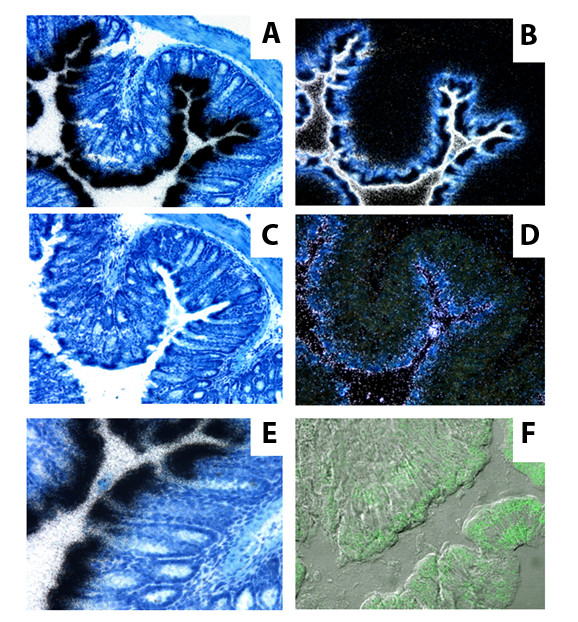
**Intestinal SAA expression and secretion**. SAA1/2 RNA, represented by the black signal in A and E, or the white signal in the counter-stained image B, was readily detectable in cross-sections of colonic epithelia (A, B), and was mainly located at the villous tips (E). SAA3 message showed similar distribution, but was much weaker (black signal in C, white in counter-stained image D). SAA immunostaining (green signal) showed a more diffuse pattern than SAA RNA, with SAA protein expressed along the crypt-villus axis (F), perhaps reflecting secretion of SAA by intestinal epithelial cells into the apical and basolateral milieu. A,C and E represent bright-field images, B and D dark-field images.

**Figure 2:**
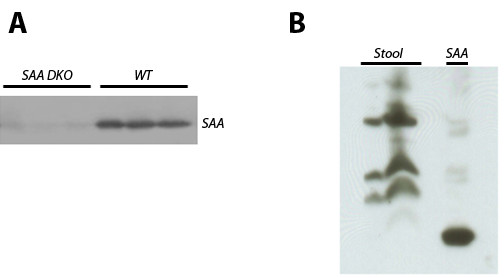
**SAA levels in plasma and stool of knockout and wildtype mice**. Samples of acute phase plasma, obtained 24 h after intravenous injection of 1 microgram LPS into wildtype mice or DKO mice (A), or extracts of stool samples from wildtype mice (B) were analyzed by immunoblotting with anti SAA1/2 antiserum following SDS-PAGE. The right lane in "B", labeled "SAA", is a positive control consisting of acute-phase plasma obtained as described for panel A. The reduced mobility of SAA in stool samples is likely caused by self-aggregation of SAA.

Sections of mouse colon were incubated with an RNA probe recognizing both *Saa*1 and -2 to determine whether SAA is expressed in mouse intestinal epithelium. Strong staining was observed at the level of the epithelium (Figure 1A,B), with most of the signal present at the tip of the villi (Figure 1E). Expression of the SAA3 isoform was weaker, but overlapped with the expression of SAA1/2 (Figure 1C,D). Immunostaining confirmed the presence of SAA in the mouse intestine, though the pattern was more diffuse (Figure 1F), perhaps consistent with release of SAA into apical and basolateral sides of the epithelium. To test whether SAA was indeed apically secreted into the lumen, extracts of fecal excrements from wildtype and SAA-knockout mice were analyzed by immunoblotting after SDS-polyacrylamide electrophoresis. As shown in Figure 2B, immunoreactivity was indeed observed in fecal extracts, consistent with secretion of SAA into the gut. Thus, SAA is expressed in the intestinal epithelium, and is secreted into the gut lumen.

**Figure 3:**
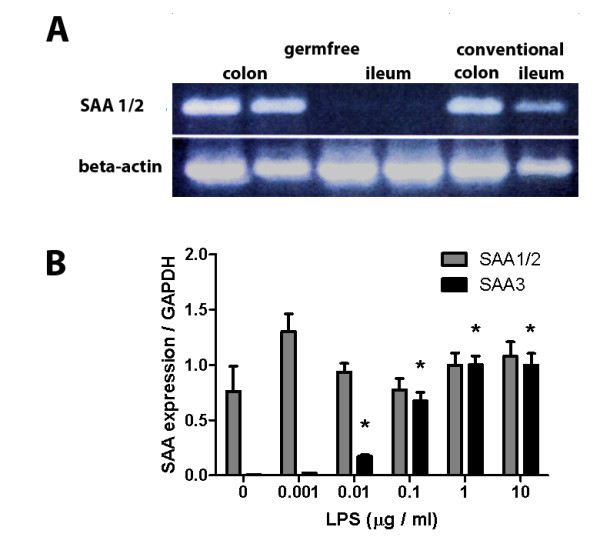
**SAA expression in mouse colon and ileum and in response to LPS**. RNA was isolated from the colon and ileum of germfree or conventionally raised mice, and cDNA was amplified with oligonucleotides recognizing a common region of SAA1/2 (A). In conventional mice, SAA1/2 was detected in colon and ileum, whereas in germfree mice, SAA1/2 was only detectable in the colon. In (B), CMT93 cells were incubated with the indicated amounts of LPS for 16 h, and SAA1/2 and SAA3 expression were determined with realtime PCR. Whereas SAA1/2 message remained rather constant, SAA3 expression markedly increased with increased LPS exposure. Each group contained data on 4 wells. Shown are average ± S.E.M. The asterisk indicates statistically significant differences (P < 0.05) compared with un-induced cells (ANOVA).

To confirm intestinal expression of SAA, RNA was extracted from intestinal segments, and cDNA was analyzed by PCR with primers recognizing a common fragment of mRNA encoded by *Saa1 *and *2*. As shown in Figure 3A, strong signal was detected in the colon, with weaker signals in the ileum. This finding could suggest a link between bacterial load and SAA expression. To test this possibility, intestinal tissue was obtained from germfree mice and analyzed for expression of SAA1/2. Surprisingly, whereas expression was nearly absent from the ileum, significant message was still detectable in the colons of germfree mice, suggesting that colonic expression is constitutive and may be independent of the presence of bacterial factors. To further test regulation of SAA expression in intestinal epithelial cells as a function of bacterial load, we used CMT93 cells, a mouse rectal epithelial line. Whereas SAA1/2 expression did not increase with increasing exposure to LPS and was already high without added LPS, SAA3 expression appeared to be strongly inducible (Figure 3B).

### Intestinal epithelial SAA expression reduces bacterial growth

**Figure 4:**
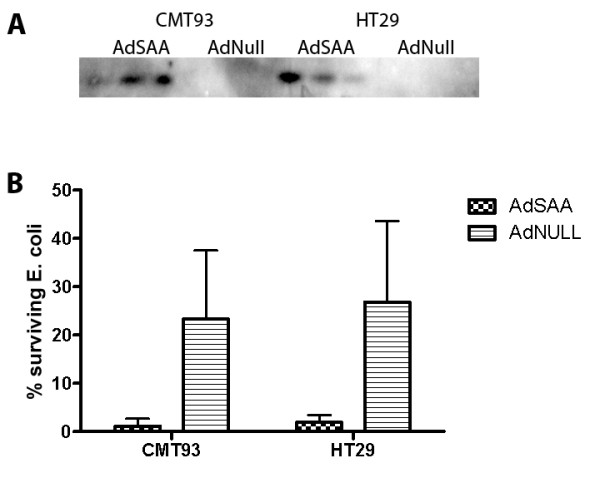
**SAA over-expression in cultured IEC reduces viability of co-cultured E. coli**. CMT93 or HT29 cells were grown on plastic supports until confluency, in medium without antibiotics. The cells were then incubated with ~20 multiplicities of infection of AdNull or AdSAA, and 48 h later, 100 cfu E. coli were added per cell for 2 h at 37°C. A small amount of medium was removed to detect SAA expression by Western blotting (A). After the 2 h incubation, serial dilutions of the culture media were grown on LB-agar. The Y-axis shows the fraction (%) of surviving bacteria relative to the positive controls (bacteria grown in cell-free medium). Show are averages ± S.D. of triplicate wells per group of a typical experiment that was conducted three times with similar outcome. The difference between AdSAA and Adnull treated groups was statistically significant (t-test; P < 0.05).

SAA expression has been shown to facilitate the killing of Gram-negative bacteria by phagocytes [15]. Since SAA is highly expressed in the intestinal epithelium, we hypothesized that SAA also affects the viability of gram-negative bacteria cultured in proximity of intestinal epithelial cells. To test this hypothesis, SAA expression in CMT93 and HT29 cells was stimulated by transduction with AdSAA whereas controls received AdNull. After 48 h, the cells were exposed for 2 h to *E. coli *DH5α (~100 bacteria per epithelial cell), and residual bacterial viability was assessed by plating serial dilutions of the conditioned media on LB agar. As shown in Figure 4, SAA over-expression significantly decreased bacterial viability in the co-culture experiments, for both cell lines.

### SAA protects against DSS colitis

**Figure 5:**
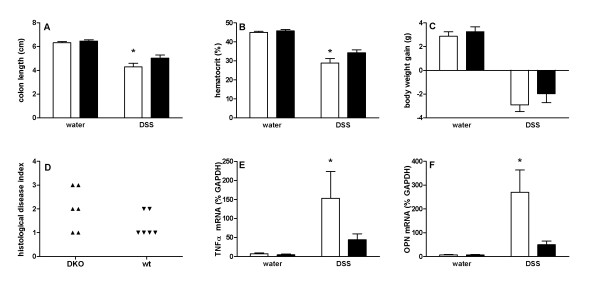
**SAA protects from experimental colitis**. SAA knockout mice (n = 7; white bars) or age and sex-matched controls (n = 7; black bars) were treated with 3% DSS in their drinking water for 6 days, while control mice (n = 5 per group) received normal water. Colon length (A), hematocrit (B), bodyweight gain (C), and histological disease index (D) were determined on day 8. Only differences in colon length and hematocrit values were statistically significant (P < 0.05, ANOVA). Real-time PCR analysis of colon tissue samples revealed significant increases in expression of TNFα and osteopontin (OPN) in colon tissue samples of DSS-treated DKO mice (E and F respectively; asterisks indicate significant differences with other groups (ANOVA and post-hoc analysis; P < 0.05)).

Failure to limit bacterial load at the intestinal epithelium increases the risk for inflammatory bowel disease, and since SAA might contribute to clearance of E. coli from the epithelium, we hypothesized that lack of SAA would increase the susceptibility to experimental colitis. To test this hypothesis, DKO mice and wildtype littermates were exposed to 3% DSS in their drinking water for one week, and colitis severity was determined. Figure 2A shows absence of SAA immunoreactivity in plasma of DKO mice after injection with bacterial LPS, illustrating that the *saa1 *and *saa2 *genes were successfully inactivated. As shown in Figure 5, DKO mice lost more weight, had increased histological disease scores, lost more blood as reflected by a decrease in hematocrit, and showed increased colon shortening compared to controls. However, only the differences in colon shortening and hematocrit values reached statistical significance. Pronounced differences were observed in the expression of TNFalpha and osteopontin in the colons of DSS-treated mice. DSS-treated DKO mice showed substantially increased TNFalpha and osteopontin mRNA levels compared to wildtype controls. Non-DSS treated mice showed little expression of these pro-inflammatory cytokines, regardless of genotype.

### SAA expression in Crohn's Disease

**Figure 6:**
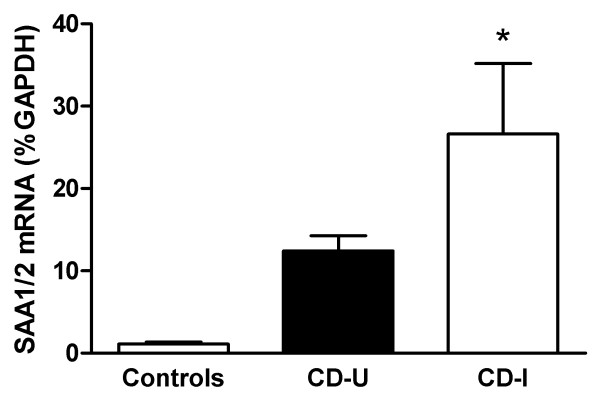
**SAA expression in human biopsies**. RNA was isolated from biopsies obtained from inflamed ileal and colonic biopsies from CD patients (CD-I ("involved")) and from uninvolved areas ("CD-U"), as well as from control patients (n = 5). Shown is the average of SAA1/2 expression ± S.D., expressed as% GAPDH. The differences between CD-I and controls were statistically significant (ANOVA; P < 0.05).

In light of the possible involvement of SAA in DSS colitis, we tested whether intestinal SAA expression is altered in inflamed and uninflamed tissue from Crohn's Disease patients versus non IBD controls. Inflamed tissue of CD patients showed significantly increased SAA1/2 expression as compared to controls and to uninvolved tissue. There was no statistically significant difference in gene expression between tissue from controls and from uninvolved areas of CD patients (Figure 6).

## Discussion

We observed that SAA1/2 and SAA3 are expressed in the intestinal epithelium, and that SAA protein is secreted into the gut lumen. Mice lacking functional SAA1/2 expression appeared to be more susceptible to DSS-induced colitis, which suggests that SAA production and secretion play a role in intestinal immune homeostasis. In vitro experiments showed that intestinal-epithelial SAA expression reduced the viability of co-cultured E. coli, suggesting that SAA may aid in the local clearance of bacteria. The expression of SAA 1/2 was increased in intestinal tissue biopsies of Crohn's Disease patients, suggesting that SAA is involved in the disease.

One of the major of higher organisms is the interaction with omnipresent bacteria. This is particularly important at mucosal surfaces, such as the intestinal epithelium, which can be colonized by large numbers of bacteria. The seemingly peaceful cohabitation of bacteria with their host represents a state of "controlled inflammation", in which the bacteria are prevented from gaining access to the body proper by an array of protective mechanisms. These include tight-junctions between epithelial cells [30], a layer of mucus covering the epithelium [19], secretion of Immunoglobulin-A [20], and an array of bactericidic proteins, enzymes, and peptides [19,21-24]. Deficiencies in one or more of these mechanisms may cause the intestinal microflora to adopt an unfavorable species composition or increases the risk of penetration by bacteria of the intestinal mucosa. Inflammatory bowel disease pathogenesis is indeed related to mutations in mechanisms allowing sensing and killing of bacteria [25,26,31].

We confirm previous reports of intestinal-epithelial expression of acute-phase SAA [17,32,33]. We also observed significant expression of SAA3 in IEC of healthy mice, in agreement with recently published data [34]. The presence of SAA3 in the epithelia is interesting, since this isoform was thought to be mainly expressed in macrophages [35]. Intuitively, since SAA is generally considered pro-inflammatory [12-14,36], one would predict that lack of SAA would result in less severe DSS colitis. We have previously demonstrated that lack of another pro-inflammatory factor, osteopontin, protects from DSS-induced colitis [28]. And yet, the DKO mice appeared to be more susceptible to DSS-induced colitis. One possible explanation is that SAA could help reduce bacterial growth near the intestinal epithelium. Recently, it was shown that plasma SAA interacts with E. coli, presumably by binding to OmpA (outer membrane protein A) in the cell wall, thereby facilitating internalization and destruction of the bacteria by neutrophils and macrophages [15]. We observed that SAA overexpression in IEC significantly reduces survival of co-cultured E. coli. We are currently investigating the mechanism for the bactericidal effect of SAA. One possibility is that, as with neutrophils, intestinal epithelial cells internalize E. coli opsonized by SAA. IEC are capable of generating bacteriocidic reactive oxygen species [37], and are also able to internalize E. coli via phagocytosis [38]. We [39] and others [40-43] have demonstrated significant expression of a candidate phagocytic receptor, Scavenger Receptor BI (SR-BI), on the apical surface of IEC. In non-professional phagocytes, such as HEK-293 cells, SR-BI strongly promotes phagocytosis [44]. As our group has shown, SR-BI also acts as a receptor for SAA [45], and we are currently investigating the role of SR-BI/SAA in the phagocytosis of bacteria and uptake of LPS into IEC. Together, these data suggest a model in which intestinal epithelial expression of SAA protects from colitis by reducing bacterial load. We also observed that intestinal biopsies from Crohn's Disease patients showed increased SAA expression. Crohn's Disease is related to local defects in bacterial sensing and killing mechanisms [25,26,31], and we speculate that SAA is upregulated in Crohn's Disease in an attempt to compensate for these defects and to protect the intestinal tissue. Detailed studies are required to test this hypothesis.

We have previously shown that DSS-induced colitis leads to a strong increase in circulating levels of acute-phase SAA [27,28], which is likely mainly derived from the liver. DSS-colitis is characterized by destruction of the intestinal epithelium, and is therefore expected to result in significant translocation of bacteria into the portal circulation. The first major organ that would encounter translocated gut bacteria is the liver. Thus, SAA would not merely be a marker for inflammation, but it could be secreted by the liver in large amounts in order to stem the spreading of translocated Gram-negative bacteria. Therefore, intestinal epithelial and hepatic SAA could act as a complementary, two-pronged mechanism of defense against intestinal Gram-negative bacteria.

## Conclusions

We have demonstrated that SAA, and likely intestinal epithelial SAA, protects from experimental colitis. At least in vitro, intestinal epithelial SAA expression greatly reduces the viability of co-cultured E. coli.

## Competing interests

The authors declare that they have no competing interests.

## Authors' contributions

EE, JW and JZ performed the majority of the experiments. RA and VA provided human tissue samples and measured gene expression in these samples. SG and YW performed in vitro experiments. MdB and FdB generated mice. EE, JW and WdW wrote the manuscript. All authors have read and approved the manuscript.

## Pre-publication history

The pre-publication history for this paper can be accessed here:

http://www.biomedcentral.com/1471-230X/10/133/prepub
